# Helminth infections in domestic dogs from Russia

**DOI:** 10.14202/vetworld.2016.1248-1258

**Published:** 2016-11-15

**Authors:** T. V. Moskvina, A. V. Ermolenko

**Affiliations:** 1 Department of Biodiversity and Marine Bioresources, Far Eastern Federal University, School of Natural Sciences, 690922 Vladivostok, Russia; 2 Department of Zoological, Laboratory of Parasitology, Institute of Biology and Soil Science, Far-Eastern Branch of Russian Academy of Sciences, 690022 Vladivostok, Russia

**Keywords:** dog, helminth infections, Russia, zoonosis

## Abstract

Dogs are the hosts for a wide helminth spectrum including tapeworms, flatworms, and nematodes. These parasites affect the dog health and cause morbidity and mortality, especially in young and old animals. Some species, as *Toxocara canis*, *Ancylostoma caninum*, *Dipylidium caninum*, and *Echinococcus* spp. are well-known zoonotic parasites worldwide, resulting in high public health risks. Poor data about canine helminth species and prevalence are available in Russia, mainly due to the absence of official guidelines for the control of dog parasites. Moreover, the consequent low quality of veterinary monitoring and use of preventive measures, the high rate of environmental contamination by dog feces and the increase of stray dog populations, make the control of the environmental contamination by dog helminths very difficult in this country. This paper reviews the knowledge on canine helminth fauna and prevalence in Russia. Practical aspects related to diagnosis, treatment, and control of parasitic diseases of dogs in Russia are discussed.

## Introduction

Dogs are the most popular pets worldwide and are infested with many parasites, which may represent a health risk for humans, especially children, the elderly and the immune-compromised [1-3]. For instance, *Dipylidium caninum*, *Echinococcus granulosus*, *Ancylostoma* spp., and *Toxocara canis* are common parasites of dogs that can affect humans in different countries around the world. Knowledge about parasite species in domestic dogs, prevalence and intensity of helminth infestations in dog populations, transmission of canine parasites and the seasonal dynamics of parasite infestations are essential for control and prevention of helminthosis in domestic animals and humans.

Investigation of free-roaming dog populations as part of urban ecology is a major key for the solution of many ecological problems in industrial ecosystems [4,5].

In Russia, 40-50% of household owners keep a dog, and the total dog population amounts to 30 million [6]. Therefore, the growing number of owned dogs in urban areas in Russia has also been accompanied by substantial increases in the stray dog population. For example, the stray dog population amounts to 12,300 specimens in Kazan [7], 9500 in Novosibirsk (http://laboratorium.narod.ru/gelm.html) and 10,000 in Omsk [8]. Stray dogs do not receive medical attention and never, or rarely, receive anti-parasitic treatments. Thus, they represent a major source of spread of gastrointestinal helminth eggs, which are harmful for people [9,10]. This article is a compendium on helminth infestations in dogs in the Russian Federation, with particular focus on zoonoses.

## The Russian Federation

The Russian Federation is the largest country in the world, covering 7,125,200 km^2^ (6,612,100 sq. miles). Russia consists of seven basic climate zones. The humid continental climate predominates in all parts of the country: European Russia, in the south of West Siberia and in the south of the Russian Far East, including the cities of Moscow and Saint Petersburg, except for the tundra and the extreme southeast.

**Figure-1:**
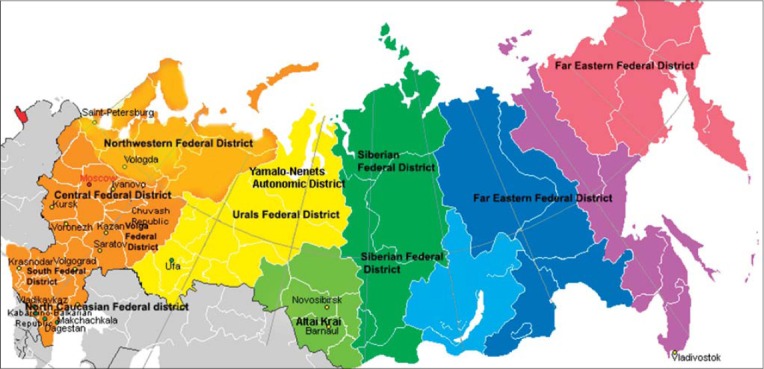
Map of researched area.

The Russian Federation is divided into eight large Federal Districts: Northwestern, South, Central, Volga, North Caucasian Federal district, Siberian, Urals, and Far East Federal District. There is lack of data about canine helminth communities and prevalence. The North Caucasian District [11-17] and Central Federal District [18-25] are the most commonly investigated areas. Sporadic reports are published from the Siberian [26-28], Volga [29-31], Ural [32], and Northwest districts [33]. A single report was published from the Far East Federal District [34] in the last 10 years (Tables-1-3 and Figure-1). The greatest number of helminth species was registered in the North-Caucasian Federal District, followed by the Volga Federal District (17 species and 11 species, respectively).

**Table 1:** Fauna of gastrointestinal helminths of domestic dogs in Russia.

Phylum	Class	Order	Family	Species	Method	Region
Platyhelminthes	Trematoda	Plagiorchiida	Opistorchiidae	*M. bilis*	AU	North Caucasian Federal District
				*M. xanthosomus*	AU	North Caucasian Federal District
				*O. felineus*	AU	North Caucasian Federal District, Siberian Federal District, Siberian Federal District, Ural Federal District
				*C. sinensis*	AU	Far Eastern Federal District
			Dicrocoeliidae	*D. lanceatum*	AU	North Caucasian Federal District
		Strigeidida	Diplostomatidae	*A. alata*	AU	Central Federal District, Siberian Federal District, Volga Federal District, North Caucasian Federal District
		Echinostomida	Echinostomatidae	*E. perfoliatus*	AU	Volga Federal District
	Cestode	Cyclophyllidea	Dipylidiidae	*D. caninum*	AU; CE	North Caucasian Federal District, Central Federal District, Siberian Federal District, Ural Federal District, Volga Federal District, Siberian Federal District, Far East Federal District
				*E. granulosus*	AU; CE	North Caucasian Federal District, Ural Federal District, Siberian Federal District, Volga Federal District
			Mesocestoididae	*M. lineatus*	AU	North Caucasian Federal District, Central Federal District
			Taeniidae	*T. multiceps* (syn. *M.multiceps*)	AU; CE	Siberian Federal District, North Caucasian Federal District, Siberian Federal District
				*T. hydatigena*	AU; CE	North Caucasian Federal District, Central Federal District, Siberian Federal District, Kazakhstan, Volga Federal District
				*T. ovis*	AU	North Caucasian Federal District
				*T. pisiformis*	AU	North Caucasian Federal District, Central Federal District
		Pseudophyllidea	Diphyllobothriidae	*D. latum*	AU, CE	Central Federal District, Urals Federal District, Volga Federal District, Ural Federal District
Nematoda	Secernentea	Ascaridida	Ascarididae	*T. canis*	AU, CE	Central Federal District, North Caucasian Federal District, North-West Federal District, Volga Federal District, Siberian Federal District, Far East Federal District, Urals Federal District
				*T. leonina*	AU, CE	North Caucasian Federal District, Central Federal District, Siberian Federal District, Volga Federal District, Ural Federal District, Far East Federal District
		Rhabditida	Strongyloididae	*S. stercoralis*	AU	Central Federal District
		Strongylida	Ancylostomatidae	*A. caninum*	AU, CE	Siberian Federal District, Volga Federal District, North-Caucasian Federal District, Central Federal District, Far East Federal District
				*U. stenocephala*	AU, CE	Ural Federal District, Volga Federal District, Far East Federal District, North-Caucasian Federal District, Central Federal District, Siberian Federal District
		Trichurida	Trichuridae	*T. vulpis* (syn *T. vulpis*)	AU, CE	North Caucasian Federal District, Central Federal District

AU=Autopsy method, CE=Coproscopically examination method, *M. bilis*=*Methorchis bilis*, *M. xanthosomus*=*Methorchis* *xanthosomus*, *O. felineus*=*Opisthorchis felineus*, *C. sinensis*=*Clonorchis sinensis*, *D. lanceatum*=*Dicrocoelium* *lanceatum*, *A. alata*=*Alaria alata*, *E. perfoliatus*=*Echinochasmus perfoliatus*, *D. caninum*=*Dipylidium caninum*, *E. granulosus*=*Echinococcus granulosus*, *M. lineatus*=*Mesocestoides lineatus*, *T. multiceps*=*Taenia multiceps*, *T.* *hydatigena*=*Taenia hydatigena*, *T. ovis*=*Taenia ovis*, *T. pisiformis*=*Taenia pisiformis*, *D. latum*=*Diphyllobothrium* *latum*, *T. canis*=*Toxocara canis*, *T. leonina*=*Toxascaris leonina*, *S. stercoralis*=*Strongyloides stercoralis*, *A. caninum*=*Ancylostoma caninum*, *U. stenocephala*=*Uncinaria stenocephala*, *T. vulpis*=*Trichuris vulpis*, *T.* *vulpis*=*Trychocephalus vulpis*, *M. multiceps*=*Multiceps multiceps*

**Table 2:** Prevalence and intensity (min and max intensity rates or mean intensity) data of dogs’ gastrointestinal helminths based on autopsy examination.

Region	Dagestan [16]	Kursk [21]	Altai [27]	North Caucasus [17]	Voronezh [20]	Caucasian mineral waters [14]	Ivanovo [18,19]	Kabardino- Balkarian Republic [12]	Moscow [24]
Total number of investigated dogs	n=320	n=67	n=72 dogs+826 fecal samples	n=35	n=12	n=385	n=173	n=17	n=86
*D. lanceatum*	9.3% 12.4±1.3	-	-	-	-	-	-	-	-
*A. alata*	8.7% 2.6±0.2	-	2.18%	16.5%	18.2%	11.1% 9-12	20.2%	29.4%	6.6% 12.6 for 1.5-3 years old dogs
*T. hydatigena*	66.5% 5.7±0.5	-	2.66%	-	-	20.2% 3-5	5.2%	29.4%	-
*E. granulosus*	66.8% 203.8±1.4	-	1.09%	80-100%	-	34.6% 11-246	-	76.5%	-
*T. ovis*	16.5% 2.0±0.1	-	1.45%	-	-	-	-	35.3%	-
*M. lineatus*	13.7% 2.4±0.2	-	-	-	-	-	1.7%	23.5%	-
*D. caninum*	26.2% 4.1±0.3	10.4%	38.01%	26% 12.8	72.7%	34.2% 5-33	68.2%	61.5%	100% 5.8-19.8 (in dogs aged 1-6 months; 7-12 months and dogs 1.5-3 years old)
*T. canis*	81.8% 39.4±0.4	38.8%	43.95%	30.5% 12.8	-	72.2% 6-49	53.7%	70.6%	6.6-100% 1.5-38.8 (in dogs aged 1-6 months; 7-12 months and dogs 1.5-3 years old)
*T. leonina*	57.5% 12.6±0.8	7.46%	39.95%	-	-	35.8% 3-19	22.5%	41.2%	100% 8.8-189 (in dogs aged 7-12 months and dogs 1.5-3 years old)
*A. caninum*	27.8% 23.6±1.0	-	2.06%	-	-	62.3% 7-52	12.7%	53%	50-100% 7.8-8.9 (in dogs aged 7-12 months and dogs 1.5-3 years old)
*U. stenocephala*	23.4% 19.2±1.2	-	16.34%	46.3% 4.6-5.3%	100% 18.5	30.9 8-91	57.7%	41.2%	100% 12.8-36.8 (in dogs aged 1-6 months; 7-12 months and dogs 1.5-3 years old)
*O. felineus*	6.5% 3.0±0.2	-	5.6%	-	-	-	-	-	-
*M. xanthosomus*	6.5% 4.1±0.3	-	-	-	-	-	-	23.5%	-
*T. vulpis*	-	8.95%	-	-	-	-	-	-	-
*S. stercoralis*	-	4.47%	-	-	-	-	6.3%	-	100% 18.6-22.8 (in dogs aged 1-6 month and dogs 1.5-3 years old)
*D. latum*	-	-	-	-	-	-	1.15%	-	-
*M. bilis*	-	-	-	-	-	10.1% 4-17	-	-	-
*T. pisiformis*	32.8% 3.1±0.2	2.98%	1.09%	-	-	12.5% 2-8	2.8%	15.4%	-
*Taenia multiceps*	22.5 2.1±0.2	-	0.85%	-	-	10.5% 3-8	-	35.3%	-

*M. bilis*=*Methorchis bilis*, *M. xanthosomus*=*Methorchis xanthosomus*, *O. felineus*=*Opisthorchis felineus*, *D.* *lanceatum*=*Dicrocoelium* *lanceatum*, *A. alata*=*Alaria alata*, *D. caninum*=*Dipylidium caninum*, *E. granulosus*=*Echinococcus* *granulosus*, *M. lineatus*=*Mesocestoides lineatus*, *T. multiceps*=*Taenia multiceps*, *T. hydatigena*=*Taenia hydatigena*, *T. ovis*=*Taenia ovis*, *T. pisiformis*=*Taenia pisiformis*, *D. latum*=*Diphyllobothrium latum*, *T. canis*=*Toxocara canis*, *T. leonina*=*Toxascaris leonina*, *S. stercoralis*=*Strongyloides stercoralis*, *A. caninum*=*Ancylostoma caninum*, *U. stenocephala*=*Uncinaria stenocephala*, *T. vulpis*=*Trichuris vulpis*, *T. vulpis*=*Trychocephalus vulpis*

**Table 3:** Prevalence data (%) of gastrointestinal helminths based on coproscopically examinations.

City	Total number of investigated dogs	Method	*T. hydatigena*	*D. caninum*	*E. granulosus*	Opisthorchiidae	*A. caninum*	*T. canis*	*T. leonina*	*U. stenocephala*	*T. vulpis*
Machachkala [15]	42	Fulleborn’s method	33.3	26.1	16.6	-	-	61.9	38	26.1	-
Kursk [21]	32	Fulleborn’s method	-	12.5	-	-	-	18.7	-	-	-
Voronezh [25]	587	Darling’s method	*Taenia* spp. 2.13	19.15	-	0.71	-	33.3	19.15	19.86	7.09
Barnaul [28]	1019	Fulleborn’s method; Kotelnikov- Chrenov’s method; Goryachev’s method	-	16.3	-	5.3 (n=150)	0.49	39.8	24.9	10.1	-
Kazan [31]	-	Fulleborn’s method; Kotelnikov- Chrenov’s method; Kotelnikov- Varenichev’s method	*Taenia* spp. 4.8	11.1	-	3.2	3.2	46	28.5	-	-
Vladikavkaz [11]	179	Fulleborn’s method	-	6.45	-	-	9.68	12.9	1.08	-	-
Moscow [23]	367	Floatation method	-	-	-	-	-	33.4	10.2	27.3	-
Novosibirsk [26]	3564	Fulleborn’s method; Kotelnikov- Chrenov’s method	*Taenia* spp. 0.59-1.87	4.68-9.42*	-	0.35-3.91*	-	9.38-30.38*	3.77-6.94*	1.21-1.31*	0.14-2.93*
Saratov [29]	1563	Fulleborn’s method	-	8.9	-	-	1.2	63.6	7.4	2.9	-
Krasnodar [22]	689	Fulleborn’s method	*T. pisiformis* 0.58	4.35	-	-	1.31	12.77	7.69	1.01	1.31
Vladivostok [34]	97	Fulleborn’s method; Sedimentation method	*Taenia* sp. 2.1	2.1	-	-	10.3	1.03	-	4.1	-

* Min and max rates. *T. hydatigena*=*Taenia hydatigena*, *D. caninum*=*Dipylidium caninum*, *E. granulosus*=*Echinococcus granulosus*, *A. caninum*=*Ancylostoma caninum*, *T.* *canis*=*Toxocara canis*, *T. leonina*=*Toxascaris leonina*, *U. stenocephala*=*Uncinaria stenocephala*, *T. vulpis*=*Trichuris vulpis*

### Helminthes of dogs in Russia

In Russia, dogs may be infested with a large number of helminths including cestodes, trematodes, and nematodes. The canine gastrointestinal helminth fauna includes 21 species (Table-1). There are eight species of tapeworms, from the following families: Taeniidae (4), Dipylidiidae (2), Mesocestoididae (1), and Diphyllobothriidae (1). Roundworms are made up of six species from the families Ascarididae (2), Ancylostomatidae (2), Strongiloididae (1), and Trichuridae (1). Flatworms include seven species from the families Opisthorchiidae (4), Dicrocoeliidae (1), and Diplostomatidae (1). Some species, such as *Toxascaris leonina*, *T. canis*, *D. caninum*, *E. granulosus* and *A. caninum*, are frequently found in dogs from different Russian regions (Tables-1-3). Other helminths such as *Mesocestoides lineatus* (Goeze, 1782), *Metorchis bilis* (Braun, 1890), *Metorchis xanthosomus* (Creplin, 1846), *Dicrocoelium lanceatum* (Stiles et Hassal, 1896), *Diphyllobothrium latum*, and *Strongyloides stercoralis* have only been sporadically reported in Russia. *Clonorchis sinensis* Looss, 1907 is an endemic species from the Asian Region; it was found in dogs from the Russian Far East [35]. Among *Taenia* species found in dogs from Russia, the most common parasites are *Taenia hydatigena*, followed by *Taenia multiceps*. Two species, *Taenia ovis* and *Taenia pisiformis*, have been found in the North Caucasian District only.

### Diagnosis of Canine Helminth Parasites

The diagnosis of helminth parasites affecting dogs is made by coprological examination methods, *Strongyloides* larva detection techniques and post-mortem examination. Helminth eggs are usually detected in feces by ordinary coprologycal techniques such as Fulleborn’s method and Darling’s method; these methods are present low sensitivity for some helminth species and result in the underestimation of the real prevalence of some parasites [36-38]. Some flotation and sedimentation techniques are used only in Russia. For instance, Kotelnikov-Varenichev’s and Kotelnikov-Chrenov’s methods are centrifugal flotation techniques, which have high sensitivity for many helminth species [39-42]. Goryachev’s sedimentation technique proposed for detecting *Opisthorchis* eggs is also use in some studies (Table-4) [43]. However, other coprological examination methods using worldwide are not used in Russia. For example, TF-test^®^ designed for detecting human gastrointestinal parasites [44] is frequency used for detecting helminth eggs in canine feces [45]. Some comparative study showed that the centrifugal flotation technique was more sensitive than centrifugal sedimentation and TF-test^®^ for recovery *Ancylostoma* spp., *T. canis*, *Trichuris* *vulpis* eggs in canine feces [45]. Another method is Willis technique has high sensitivity for *T. canis* eggs in canine feces [46]. Moreover, recent study showed that this method performed better than the centrifugal flotation techniques and Hoffman-Pons-Janer technique for detecting *Ancylostoma* spp. in dog feces [47].

**Table 4:** Comparison of coproscopically examination method using in Russia.

Method	Solution (Specific gravidity)	Technique	Sensitivity
Fulleborn [36]	NaCl (1.2)	Flotation	Good sensitivity for *Toxocara*, *Toxascaris* and *Trichuris* eggs which frequently appear in canine faeces
Darling [37,38]	NaCl + C_3_H_8_O_3_ (1.21)	Flotation-sedimentation	Low sensitivity for flatworms and *Diphyllobothrium* eggs identification
Goryachev [43]	NaCl (1.2)	Sedimentation	Use only for *Opisthorchis* eggs detection
Kotelnikov-Varenichev [39-41]	ZnCl_2_ (1.82)	Centrifugation flotation	High sensitivity for *Toxocara*, *Toxascaris* and *Trichuris* eggs, flatworms and cestode eggs
Kotelnikov-Chrenov [41,42]	NH_4_NO_3_ (1.28)		High sensitivity for flatworm eggs, *Taenia* eggs and nematode eggs

Traditional Baermann’s and modified Baermann-Orlov’s methods are used for *S. stercoralis* larva detection from canine feces [48,49]. Zink sulfate flotation technique which is sensitive for *S. stercoralis* larva is not used in Russia [50].

Necropsy examination is performed according to the standard procedures [49,51]; methods of total and part helminthological examination suggested by Skrjabin [52] used in parasitological study.

### Prevalence of Helminth Infections in Dogs in Russia

Data obtained from reports in different regions showed broad prevalence rate fluctuations. The prevalence depends on climate, living conditions, and quality of veterinary care [53,54]. Many reports did not include data about the total prevalence of gastrointestinal parasites in dogs. However, individual prevalence rates for different parasites were greater than 50% in 37% of studies. High prevalence rates and a broad parasite spectrum were found in studies using the necropsy method (Table-2).

Overall, nine parasites species were found in studies using coproscopic examination methods. Some species, such as *M. xanthosomus*, *M. bilis*, *S. stercolaris*, *M. lineatus* and *D. latum*, were not found on fecal examination. In some cases, it was also difficult to distinguish species of eggs Opisthorchiidae family [55] and eggs genus *Taenia* based only on morphological characters [56].

The most common species in Russia was *T. canis*, followed by *D. caninum*, *T. leonina*, and *Uncinaria stenocephala* (Tables-2 and 3). *A. caninum* was found in 52.3% of studies conducted in areas with a continental or temperate climate, where there are warm summers and high humidity, as these conditions are optimal for *A. caninum* larval development [57].

Flatworms of the genus *Metorchis* (Looss, 1899) are worldwide parasites of Cyprinidae fishes, and infest fish-eating mammals. In Russia, *M. xanthosomus* was found in Dagestan and Kabardino-Balkaria Republic, while *M. bilis* was found in Caucasian Minerals Water (Table-2). It is interesting that these species were found separately in different regions since both species have common intermediate hosts. The first host is the mollusk *Bithynia tentaculata* L. 1758 living in the Palearctic zone, except the North zone [58]. The second hosts are Cyprinidae fishes [59].

The cestode *M. lineatus* has a worldwide distribution. It has been found in Europe [60], the Middle East [61], Africa [62], North, and South America [63]. Adult worms live in small intestine of carnivorous mammals including fox, wolves, dogs, cats, coyotes, raccoons, and lynxes [64]. One case of the peritoneal larval stage was recorded in dogs from Germany [65]. This species spreads proglottids via the feces so it cannot be found with flotation methods. In Russia, dogs infested with *M. lineatus* were found in Dagestan with a low prevalence rate [16].

### Dogs and People: Problem of Parasite Zoonoses

Most parasites species found in dogs from Russia have zoonotic potential. *T. canis* is the most common canine intestinal endoparasites worldwide. Humans are infected by *Toxocara* via ingestion of embryonated eggs in contaminated soil [66]; however, pet hair can also contain embryonated eggs [67]. The first importance reports about human toxocariasis in Russia were published in 1961-1962 [68,69]. Only in 1988 did the connection between the source of toxocariasis in dogs and nosoareal of toxocariasis in humans appear [70]. Recently, the problem of toxocariasis in humans and dogs has been highlighted worldwide. In the Russian Federation, toxocariasis has frequency appeared in children, especially in children with allergic diseases (31-47% of children with allergic diseases) [71]. Ocular toxocariasis is frequently recorded in children, whereas visceral toxocariasis is more frequently recorded in adults. Since 1991, *Toxocara* infestation rates in people have increased. For example, *Toxocara* infestation was recorded in 0.03 per 100,000 people in 1991 and in 2.32 per 100,000 people in 2012.

However, toxocariasis was recorded in 2.1 per 100,000 people from 2008 to 2012 [72]. Infestation rates are broadly variable in different regions and in people of different age groups. *Toxocara* prevalence in people from Russia is 5.4% in Moscow and Tula, 7.4% in Dagestan and 6% in the Irkutsk District [73]. The *Toxocara* prevalence was 16.7% in children from the Altay Region, whereas toxocariasis was recorded 6 times more frequently in adults from the Krasnodar region. *E. granulosus* is a widespread parasite, which has a major medical, veterinarian and socioeconomic cost. The adult parasite stage occurs in the canine small intestine, and people are infested by ingestion of contaminated food [74] or by direct contact with contaminated dogs that retain eggs on their coats [75]. Echinococcosis in humans is the most serious parasitic disease, as a fatal outcome is recorded in 2-23% of cases [76]. Higher prevalence rates appear in China, western and southern Russia, Southwestern Europe, South Africa and Central and South America [76]. The Orenburgskii Region is the most problematic territory in Russia, as echinococcosis was registered in 3.4±0.4 per 100,000 people [77]. Recently, DNA-based studies showed that *E. granulosus* comprise 10 genotipes which have been distinguished in different species [78]. *E. granulosus* s. s. (G1-G3), *E. canadensis* (G6, G8 and G10) were found in Russia [79].

People are infrequently infested with *D. caninum*, which occurs through ingestion feline fleas infected with tapeworms [80,81]. *D*. *caninum* infestation was also registered in Russia. Dipylidiasis was recorded in humans from the Orenburgskii Region [82], Moscow [83], and the Kabardino-Balkarian Republik [84].

Liver flukes of the genera *Clonorchis* Loos, 1907, *Metorchis*, and *Opisthorchis* Blanchard 1895, in the family Opisthorchiidae, exploit freshwater snails and fish as the first and second intermediate hosts, respectively. The final hosts, fish-eating birds and mammals, including dogs and humans, are infected by eating fish harboring infective metacercariae [85]. Feces of dogs infested with *Opisthorchis felineus* and *Metorchis* spp. are major sources of water contamination [86]. The largest infestation center is located in the Ob-Irtysh basin and includes 10 regions of Russia and Kazachstan. The infestation rate is 51-82% in humans. The other intensive infestation center is Chulym River in the Krasnoyarsk region; the prevalence of *O. felineus* in people is 70-80% [87].

*M. bilis* is also found in fish from the Ob-Irtysh basin. The prevalence rate in people from West Siberia is 28.1% [88].

The flatworm *C. sinensis* is endemic to the Far Eastern region and it was also found in dogs and people from China and Korea [86,89]. *C. sinensis* is frequently found in people from the Russian Far East [90].

The tapeworm *M. lineatus* has major veterinary importance, and occasionally, it has been found in people [91]. In Russia, cases of *M. lineatus* infestation in humans have not been reported, however dog feces containing proglottids are major sources of environmental contamination.

*D. latum* is a common parasite of fish-eating mammals. Fecal contamination of water is a source of *D. latum* spread. In the last 10 years, only one report regarding canine infestation from Ivanovo was published. A big center of infestation located in Russia is Baikal Lake. Infestation rates in humans from the Irkutsk Region were 9.6 cases per 100,000 people [92].

Another flatworm, *Alaria alata*, has specific veterinary importance [93], however, several reports about human larval alariosis were published since 1973 [94,95]. In Russia, *A. alata* was found in dogs, foxes, wolves and badgers in the Vladimir, Ivanovo and Moscow regions, and in the Volgograd and Astrachan regions, in the North Caucasian District. Prevalence rates were 38.4-48.6% in farm dogs, 46.1-59.3% in stray dogs and 100% in wolves and foxes [96].

### Control and Prevention

Zoonosis is the major veterinary and medical problem. Zoonotic infestations include well-known parasite species such as *T. canis* and *E. granulosus*, which have a worldwide distribution.

However, there are no official guidelines for the control of endoparasite infestations in dogs, such as that provided by the Companion Animal Parasite Council (CAPC: http://www.capcvet.org/) in the United States and the European Scientific Counsel Companion Animal Parasites (ESCCAP: http://www.esccap.org/) in Europe.

There is scant information about problems of veterinary epidemiology in Russia. Two guidelines for the sanitary and veterinary rules were published by the Veterinary Department of the Ministry of Agriculture of the Russian Federation (http://docs.cntd.ru/document/1200050554) with State Sanitary and Epidemiological Supervision of the Russian Federation (http://rospotrebnadzor.ru/documents/details.php?ELEMENT_ID=2890). Veterinarians do not have a native source of information for parasite epidemiology, life cycles or control measures [97].

Parasitology monitoring is provided irregularly. Poor living conditions and lack of anti-parasitic medication causes environmental contamination with helminth eggs [98]. Environmental contamination of helminth eggs is a big problem in many urban and rural areas in Russia, especially in agricultural areas, where feces are used for fertilizing. Currently, basic methods for dog helminth infections prevention include regular deworming of domestic animals, control of environmental contamination (avoid contamination of canine feces in public places), and spread of information about zoonotic parasites [99]. Moreover, control of food quality and pet diets help to prevent parasite infestations. For example, to prevent *C. sinensis* and *O. felineus* infestation in dogs, it is recommend to avoid the feeding of fresh cyprinid fishes [99].

Many dog owners cannot afford preventive measures and will act only when a life-threatening problem is affecting their animals. Furthermore, there are a large number of free-roaming dogs populations in the Russian cities. Government is not able to manage these animals due to the lack of adequate infrastructure and trained personnel to conduct an effective long-term population control program. As a result, pet dogs and cats are usually endangered by a wide range of parasites that may cause disease in them and eventually in their human counterpart.

## Conclusions

The close contact between pets and humans may involuntarily represent a hazard for humans. Therefore, to avoid the potential risks associated with owning a pet, it is fundamental to maintain pets in good health and protect them from zoonotic pathogens.

Therefore, veterinary practitioners and medical physicians should work together toward improving the well-being and general health of both animals and humans.

## Authors’ Contributions

MTV and AVE participated in the draft and revision of the manuscript. Both authors read and approved the final manuscript.
